# Bioenergetic Changes during Differentiation of Human Embryonic Stem Cells along the Hepatic Lineage

**DOI:** 10.1155/2017/5080128

**Published:** 2017-02-06

**Authors:** Branden M. Hopkinson, Claus Desler, Mark Kalisz, Peter Siig Vestentoft, Lene Juel Rasmussen, Hanne Cathrine Bisgaard

**Affiliations:** ^1^Department of Cellular and Molecular Medicine, Faculty of Health Sciences, University of Copenhagen, Copenhagen, Denmark; ^2^Center for Healthy Aging, Faculty of Health Sciences, University of Copenhagen, Copenhagen, Denmark; ^3^Department of Developmental Biology, Hagedorn Research Institute, 2820 Gentofte, Denmark

## Abstract

Mitochondrial dysfunction has been demonstrated to result in premature aging due to its effects on stem cells. Nevertheless, a full understanding of the role of mitochondrial bioenergetics through differentiation is still lacking. Here we show the bioenergetics profile of human stem cells of embryonic origin differentiating along the hepatic lineage. Our study reveals especially the transition between hepatic specification and hepatic maturation as dependent on mitochondrial respiration and demonstrates that even though differentiating cells are primarily dependent on glycolysis until induction of hepatocyte maturation, oxidative phosphorylation is essential at all stages of differentiation.

## 1. Introduction

Mutations of the mitochondrial polymerase gamma cause a mitochondrial DNA (mtDNA) mutator phenotype which in mice has been correlated to premature aging and decreased lifespan [1, 2]. The progeroid phenotype is demonstrated to be primarily elicited through the effect of mitochondrial dysfunction on stem cells, rather than on postmitotic cells [3]. The purpose of this work is to provide a detailed understanding of the need of mitochondrial function throughout differentiation of stem cells in order to better understand the relationship between dysfunctional mitochondria, stem cell differentiation, and premature aging.

The human mtDNA exclusively encodes 24 peptides needed for mitochondrial protein synthesis and 13 essential subunits of the mitochondrial electron transport chain (ETC) and ATP synthase. Together they produce ATP by oxidative phosphorylation in an aerobic process. ATP can also be produced by glycolysis in an anaerobic process. The preference of cells for oxidative phosphorylation or glycolysis is determined by cell type, state of the cell, and the availability of oxygen. Oxidative phosphorylation is more efficient in producing ATP when compared to glycolysis. Conversely, glycolysis has the ability to produce ATP at a higher rate than mitochondrial respiration and can provide more carbon intermediates necessary for biosynthesis (reviewed in [4]).

Embryonic stem cells (ESC) have a preference for glycolysis [5, 6]. During differentiation, however, changes of mitochondrial morphology indicate a fluctuating preference towards oxidative phosphorylation. During this process mitochondrial networks expand to adopt the more sporadic cytoplasmic configuration seen in typical somatic cells within differentiated tissue [5, 7]. Furthermore, there is an increase in copy-number of mtDNA encoding subunits of the ETC [8]. As a result, stem cells increase their oxygen consumption 10-fold in the course of differentiation [7].

The flux between glycolysis and oxidative phosphorylation not only is a marker of differentiation status but is also essential for stem cell fate. Suppression of oxidative phosphorylation retards differentiation of stem cells [9, 10], while inhibition of glycolysis halts proliferation and promotes cell death [6]. Furthermore, during dedifferentiation, or upon induction of pluripotency, mitochondrial morphology reverts to that of stem cells displaying immature spherical and cristae-poor structures [11, 12], and, in itself, promotion of glycolysis at the expense of oxidative phosphorylation promotes dedifferentiation [12].

While mitochondrial dysfunction in stem cells has been demonstrated to result in a progeroid phenotype [3], the underlying mechanism is largely unknown. In this study, the cellular utilization of oxidative phosphorylation and glycolysis is determined at specific time points in the course of a 20-day differentiation protocol of stem cells of human embryonic origin along the hepatic lineage. This study highlights especially the transition from hepatic specification to hepatic maturation as dependent on mitochondrial respiration. Together, this will help elucidating the role of mitochondria in the regulation of stem cells and progeria.

## 2. Materials and Methods

### 2.1. Materials

The human embryonic stem cell line BG01V/hOG (hESC) can be purchased from Thermo Fisher Scientific (http://www.thermofisher.com). This cell line is a variant derived from the BG01V hESC line genetically modified to express Emerald Green Fluorescent Protein (EmGFP) under the Oct4-promotor wherefore visual inspection of the GFP expression can be used to verify pluripotency [13].

All media and supplements were obtained from Thermo Fisher Scientific or Sigma-Aldrich (http://www.sigmaaldrich.com) unless otherwise stated. Antibodies were acquired from R&D Systems (http://www.rndsystems.com), Dako (http://www.dako.com), Santa Cruz Biotechnology (http://www.scbt.com), Abcam (http://www.abcam.com), and Thermo Fisher Scientific.

### 2.2. Cell Culture

The BG01V/hOG cell line was cultured on mitotically inactivated mouse embryonic fibroblasts (MEF) in hESC media containing DMEM/F-12 media (1 : 1) supplemented with GlutaMAX™, 20% Knockout™ Serum Replacement, nonessential amino acids, 2-mercaptoethanol at 0.1 M, basic fibroblast growth factor at 4 ng/mL (R&D Systems), and penicillin/streptomycin at 100 units/mL. Cells were cultured at 37°C under atmospheric O_2_ and 5% CO_2_.

### 2.3. Cell Differentiation

**Figure 1:**
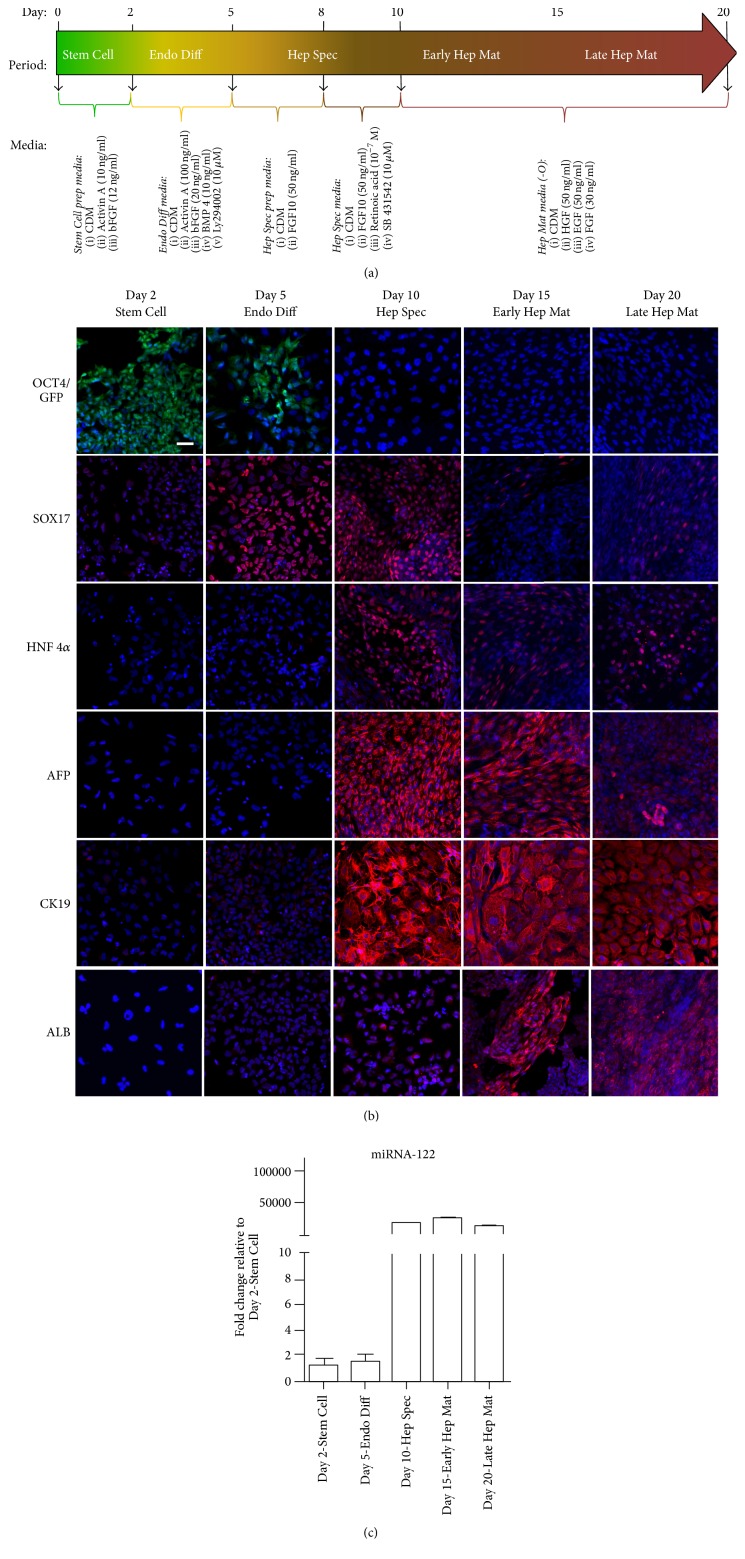
(a) Schematic presentation of the differentiation protocol [14] used to measure the bioenergetics in the course of BG01V/hOG (hESC) line differentiation along the hepatic lineage. (b) Expression of the pluripotency marker OCT4 visualized by detection of EmGFP (in green) and immunofluorescence detection of the definitive endoderm marker SOX17 and the hepatic differentiation markers HNF4*α*, AFP, CK19, and albumin, all shown in red, in the course of BG01V/hOG (hESC) line differentiation along the hepatic lineage. Nuclei are highlighted with DAPI staining shown in blue. Scale bars represent 50 *μ*m (50 microns). (c) Expression profile of miRNA-122 detected by real-time RT-PCR in the course of BG01V/hOG (hESC) line differentiation along the hepatic lineage.

**Figure 2:**
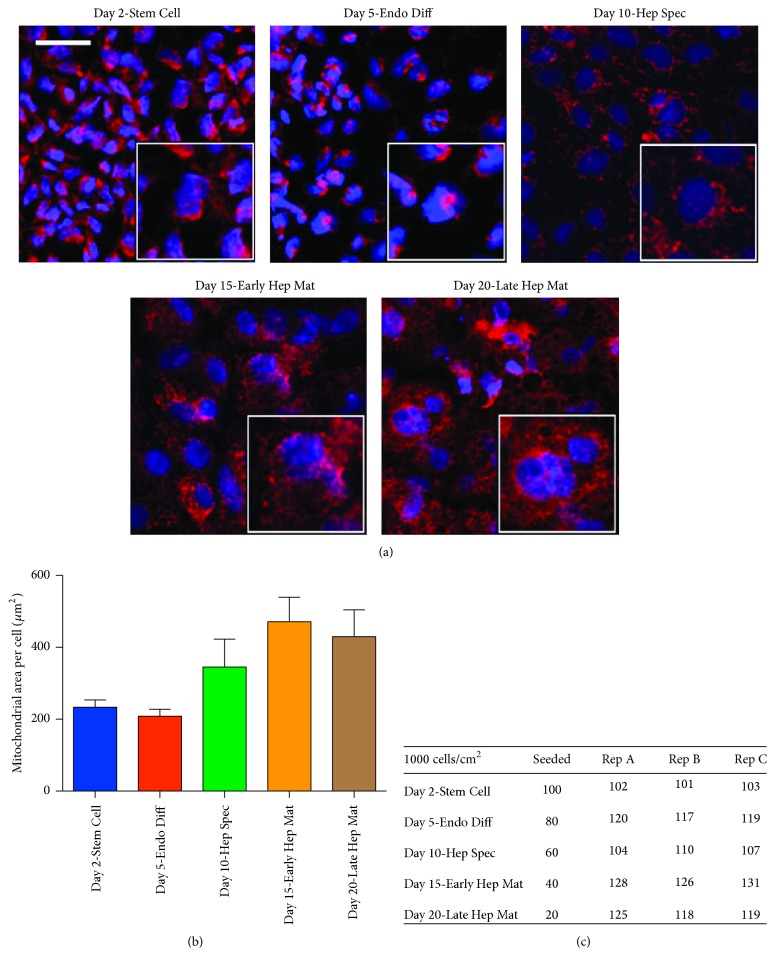
(a) Immunofluorescence detection of COX4 visualized in red in the course of BG01V/hOG cell differentiation along the hepatic lineage. Nuclei are highlighted with DAPI staining shown in blue. Scale bars represent 50 *μ*m (50 microns). (b) Quantification of cellular areas covered by mitochondrial networks in the course of BG01V/hOG cell differentiation along the hepatic lineage. (c) To ensure equal cell numbers of approximately 100,000 cells/cm^2^ at harvest for bioenergetics analyses cells were seeded at different densities and cell numbers counted at harvest as shown in (c).

Prior to differentiation the BG01V/hOG cells were passaged to a feeder-free system composed of mTeSR®1 media (STEMCELL Technologies, http://www.stemcell.com) and Matrigel™ (BD Biosciences, http://www.bdbiosciences.com) coated plates. Cells were passaged at least twice in this system to remove mitotically inactivated MEF from the BG01V/hOG cell culture. For differentiation along the hepatic lineage, BG01V/hOG cells were passaged to plates coated with 0.1% porcine gel. The cells were then subjected to 20-day media regime consisting of daily media changes (Figure 1(a)) as described in Touboul et al. [14]. For indirect immunofluorescence analyses BG01V/hOG cells were seeded in 48-well plates (Corning Life Sciences, http://www.corning.com) at 21,000 cells/cm^2^. For determination of glycolysis and mitochondrial respiration BG01V/hOG cells were seeded in Seahorse XF24 cell culture microplates (Seahorse Bioscience, http://www.agilent.com) at various densities to adjust for cellular growth through differentiation. Cell plates harvested at different time points were at day 0 seeded in following number: Day 2 (100,000 cells/cm^2^), Day 5 (80,000 cells/cm^2^), Day 10 (60,000 cells/cm^2^), Day 15 (40,000 cells/cm^2^), and Day 20 (20,000 cells/cm^2^). Three wells were trypsinized from Seahorse microplates prior to analyses and cells were counted to ensure that the number of cells per Seahorse well at harvest was approximately 100,000 cells/cm^2^ (Figure 2(c)). In brief, cells for differentiation were cultured in a chemically defined media (CDM) supplemented with small molecules and growth factors. The composition of CDM was as follows: Iscove's Modified Dulbecco's Medium and Ham's F12 Medium both with GlutaMAX at a 1 : 1 ratio supplemented with chemically defined lipid concentrate at 1x, penicillin/streptomycin at 100 units/mL, bovine serum albumin fraction V at 5 mg/mL, monothioglycerol at 450 *μ*M, holotransferrin at 15 *μ*g/mL (R&D Systems), and insulin at 7 *μ*g/mL. Cells were kept in an incubator at 37°C under atmospheric O_2_ and 5% CO_2_. At the time points indicated in Figure 1(a), the composition of the medium was altered by addition of the specified small molecules and growth factors to support a specific period in the differentiation process along the hepatic lineage. Plates were harvested for analyses at the following time points: Day 2: Stem Cell, after 2 days' culturing in Stem Cell media; Day 5: Endo Diff, after 3 days' exposure in definitive endoderm induction media; Day 10: Hep Spec, after 5 days' induction in hepatic specification media; Day 15: Early Hep Mat, after 5 days' induction in hepatocyte maturation media; and finally Day 20: Late Hep Mat, after 10 days' induction in hepatocyte maturation media.

### 2.4. Indirect Immunofluorescence Analysis

Differentiating BG01V/hOG cells were fixed in paraformaldehyde (4%) for 15 min at room temperature (RT) on Days 2, 5, 10, 15, and 20, respectively. Plates were stored at 4°C until all time points were collected (maximum 20 days) and analysed collectively under identical conditions. Samples were permeabilized with Triton X-100 (0.5%) for 20 min at RT, before incubation with blocking solution containing 10% donkey or goat serum for 1 hour at RT. Subsequently, the samples were incubated overnight at 4°C with primary antibodies followed by 30 min incubation at RT with secondary antibodies (Table 1) and finally mounted with ProLong Gold containing DAPI. The samples were imaged using a Zeiss LSM 780 laser scanning confocal microscope (the Core Facility for Integrated Microscopy, Faculty of Health and Medical Sciences, University of Copenhagen, Denmark). To establish a comparative series of images, the confocal laser used was optimized at the stage showing the peak intensity for each antibody series and microscope parameters were copied and used for that specific antibody through all plates. Stains without primary or secondary antibodies provided internal controls to establish target specificity of antibodies. For quantification of the cellular mitochondrial network areas during the course of hepatic lineage differentiation ImageJ (http://www.imagej.net) was used. The number of cells per image was assessed by nuclear DAPI staining and area of mitochondrial networks was assessed using immunofluorescence staining of the mitochondrial marker COX4. Average area of mitochondrial network per cell was calculated by dividing total area of mitochondrial network in the image by the number of nuclei in the image.

### 2.5. Real-Time RT-PCR Analyses

Real-time RT-PCR quantification of miRNA expression was carried out using TaqMan MicroRNA Assay Kits for miRNA-122 according to the manufacturer's protocol (Applied Biosystems, Life Technologies Corporation). In brief, cells were seeded at 21,000 cells/cm^2^ and induced to differentiate. Total RNA was extracted from cell cultures using TRIzol Reagent (Invitrogen, Carlsbad, CA, USA), and 10 ng of total RNA was reverse transcribed. Real-time PCR analysis was performed at 95°C for 10 min, followed by 40 cycles of 95°C for 15 sec and 60°C for 1 min. All reactions were done in triplicate.* C*_*T*_ was determined using threshold settings adjusted for miRNA-122 based on standard curves of 10-fold dilutions. The threshold value was placed in the linear phase of exponential amplification and set at a point that maximised the precision of replicates. Results were expressed as fold change in miRNA-122 expression as compared to the Day 2 time point calculated as 2^−ΔCt^.

### 2.6. Determination of Glycolysis and Mitochondrial Respiration

Mitochondrial bioenergetics of BG01V/hOG cells in the course of differentiation along the hepatic lineage were measured at the five different time points using an XF24 Extracellular Flux Analyzer (Seahorse Bioscience,). For each time point, measurements were made from 8 independent inductions. At harvest, the Seahorse plates were washed and Seahorse assay media (Seahorse Bioscience) were added, supplemented with pyruvate at 1 mM and glutamine at 2 mM, and adjusted to pH 7.6. Plates were incubated in a CO_2_-free incubator at 37°C for 1 hour to allow temperature and pH equilibration, after which oxygen consumption rate (OCR) as well as extracellular acidification rate (ECAR) of each well was measured in the XF24 Extracellular Flux Analyzer over a period of 144 minutes. For determination of oxidative phosphorylation properties, the ATP synthase inhibitor oligomycin at 0.75 *μ*M, the uncoupler carbonyl cyanide-4-(trifluoromethoxy)phenylhydrazone (FCCP) at 1 *μ*M, and complex III inhibitor antimycin A at 2 *μ*M were added to wells after 27, 63, and 108 minutes, respectively. This allowed the determination of basal ATP turnover, maximal respiration, and reserve respiratory capacity. For determination of glycolytic properties, D-glucose at 10 mM, oligomycin at 0.75 *μ*M, and the glycolytic inhibitor 2-deoxy-D-glucose (2DG) at 150 mM were added at the same time points. For determination of oxidative phosphorylation and glycolysis, OCR and ECAR measurements used for calculation were taken immediately before addition of next compound. This allowed the determination of basal rate of glycolysis and glycolytic reserve capacity.

### 2.7. Statistics

Single classification analysis of variance (ANOVA) was used to test differences between bioenergetic properties. Assumptions of normality were checked by visual inspection prior to ANOVA. When the ANOVA indicated significant differences, Tukey's honestly significant method was used to test for differences between bioenergetics properties.

## 3. Results

### 3.1. Human Embryonic Stem Cell Differentiation along the Hepatic Lineage

The 20-day differentiation protocol from Touboul el al. [14] was used to differentiate BG01V/hOG human embryonic stem cells along the hepatic lineage into hepatocyte-like cells. Cell differentiation was followed by immunofluorescent detection of verified differentiation markers in the hepatic lineage (Figure 1(b)).

As visualized by EmGFP expression and consistent with the results presented by Touboul et al., transcription of the pluripotency marker OCT4 was active at Day 2 in stem cells. At Day 5, upon 3 days' induction in definitive endoderm media, some cells were still actively transcribing OCT4. However, at the same time point a nuclear expression of the definitive endoderm marker protein SOX17, a transcription factor important in the developmental process between endoderm formation and early gut formation, was detected. The SOX17 protein expression was sustained at Day 10 following culture in the hepatic specification media for 5 days but suppressed at the later time points after culture in hepatocyte maturation media. Immunofluorescence staining of hepatocyte nuclear factor 4*α* (HNF4*α*) protein, a nuclear factor required for hepatic lineage differentiation and development of the liver, revealed low cytoplasmic expression at Days 2 and 5 and peak expression and translocation to the nucleus from Day 10 to the end of the differentiation regimen. A similar transcription profile was observed in a real-time RT-PCR analysis of miRNA-122 (Figure 1(c)). The expression of this highly liver specific microRNA is developmentally regulated and increases in the liver over the course of development constituting approximately 70% of the miRNA pool in the adult liver [15]. Interestingly, HNF4*α* has been shown to regulate the expression of miRNA-122 [16]. Therefore, the concomitant expression profiles of these two parameters strongly indicate a specification to the hepatic lineage between Day 5 and Day 10 in the presented differentiation protocol. This was further supported by the expression profiles of *α*-fetoprotein (AFP) and Cytokeratin 19 (CK19) (Figure 1(b)). *α*-Fetoprotein is a major plasma protein expressed in emerging hepatocytes during embryonic and fetal liver development but the protein cannot be detected in mature hepatocytes of normal adult liver [17]. In the present study, AFP was not expressed at Day 2 and Day 5 but expression was detected upon incubation in hepatocyte specification media at Day 10 and sustained at the same high level at Day 15 following incubation for 5 days in hepatocyte maturation medium. Similarly, expression of CK19, an intermediate filament protein possibly involved in cellular restructuring in early liver maturation, was induced at Day 10. Both AFP and CK19 have been shown to be expressed in immature hepatocytes early in liver development in humans but are differentially regulated when the fetal immature hepatocyte compartment is remodeled to form distinct populations of more mature hepatocytes and bile duct cells. Thus, while expression of CK19 is sustained in the newly formed bile duct cells, its expression is downregulated in the maturing fetal hepatocytes [17, 18]. Interestingly, a decreased intensity in expression of AFP and CK19 proteins was found at Day 20 in the maturing hepatocyte-like cells. In contrast, albumin (ALB), a major plasma carrier protein exclusively produced by hepatocytes and a well-established biomarker for functional hepatocytes, showed a gradual increase in expression upon incubation in the hepatocyte maturation medium. It is worth noting that addition of oncostatin M to induce further maturation of hepatocyte-like cells showed no effect on the parameters measured in the current study (data not shown). Collectively, our data is in concordance with Touboul et al. [14] showing that the sequential addition of growth factors and small molecules to a chemically defined medium can direct human embryonic stem cells along the hepatic lineage and result in a hepatocyte-like cell phenotype.

### 3.2. Alterations of Mitochondrial Morphology during Differentiation along the Hepatocytic Lineage

Cytochrome *c* oxidase (complex IV) is the fourth and final enzyme of the ETC and is only located in the inner mitochondrial membrane. As complex IV is the final electron acceptor of the ETC, the enzyme is essential for oxidative phosphorylation. Immunofluorescence staining of complex IV with an antibody against COX4 showed tight perinuclear clustering of the complex and thereby mitochondria in cells at Day 2 and Day 5 (Figure 2(a)). Upon 5 days' incubation in hepatic specification media, cells at Day 10 showed expansion of mitochondria in the cytoplasm with formation of thin mitochondrial networks (Figures 2(a) and 2(b)). Similar observations were made at Day 15 and Day 20 after incubation in hepatocyte maturation media.

### 3.3. Glycolytic Profiles during Differentiation along the Hepatocytic Lineage

**Figure 3:**
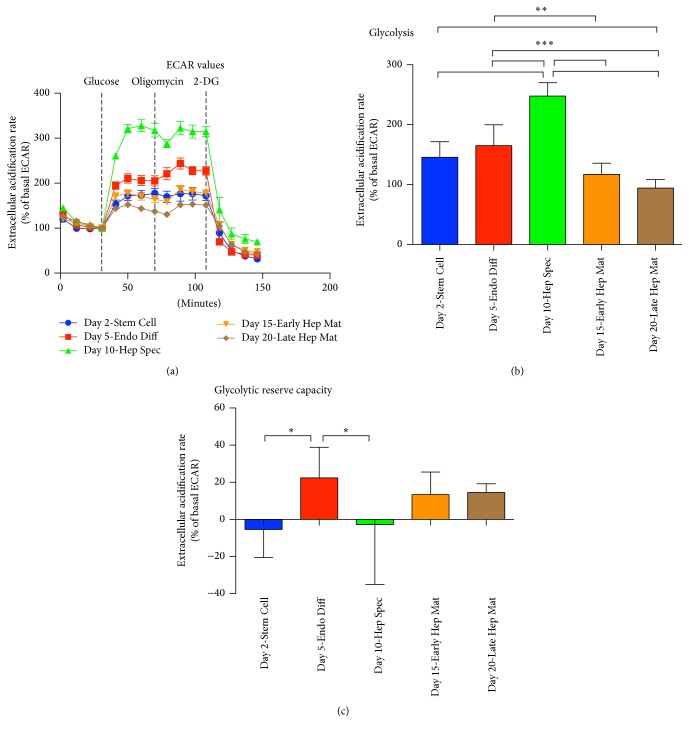
Measurement of acidification rate at five successive time points in the course of BG01V/hOG (hESC) differentiation along the hepatic lineage. (a) Parameters of glycolysis were determined according to rate of acidification after addition of glucose, oligomycin, and 2-DG. (b) Rate of glycolysis was measured as increase of acidification rate after addition of glucose. (c) Glycolytic capacity was measured after addition of glucose and oligomycin (^*∗*^*P* < 0.05, ^*∗∗*^*P* < 0.01, and ^*∗∗∗*^*P* < 0.001; *n* = 8; error bars indicate SD).

Attributes of glycolysis were measured as a result of lactate mediated acidification of media surrounding the cells. Rates of glycolysis were determined as percent increase of extracellular acidification rate after addition of glucose. At Day 2 when the cells had a stem cell phenotype, the glycolytic rate was 146%. This rate was increased at Day 5 upon incubation in endoderm differentiation media to 165% and reached a maximum of 248% at Day 10 after incubation in hepatic specification media which was significant higher than rate of glycolysis measured at other time points (*P* < 0.001, *n* = 8). Upon further differentiation along the hepatic lineage by incubation in hepatocyte maturation media, the glycolytic rate was reduced to 117% and 94% at Day 15 and Day 20, respectively (Figures 3(a) and 3(b)). This signifies an increasing requirement of ATP produced by glycolysis through the first 10 days of differentiation, reaching an absolute maximum at Day 10 after incubation in hepatic specification media. Upon incubation in hepatocyte maturation media, ATP produced by glycolysis decreases dramatically, and it is at Day 20—our latest time point harvested—no longer statistically discernable from cells that have not been exposed to glucose (Figures 3(a) and 3(b)).

Glycolytic reserve capacity is a measure of the difference in extracellular acidification rate in cells before and after treatment with oligomycin. Oligomycin inhibits the ATP synthase and thereby ATP produced by oxidative phosphorylation. Glycolytic reserve capacity therefore reflects the compensatory increase of glycolysis corresponding to the level of ATP no longer produced by oxidative phosphorylation. During the differentiation regimen, glycolytic reserve capacity is not present in stem cells measured at Day 2 and upon hepatic specification at Day 10 (Figures 3(a) and 3(c)). This indicates that, at these time points, ATP demand is almost exclusively met by glycolysis. Upon incubation in hepatocyte maturation media at Day 15 and Day 20, the glycolytic reserve capacity is increased to 14%. This increase suggests an emerging requirement of ATP produced by oxidative phosphorylation at these later time points. Interestingly, upon 3 days of incubation in endoderm differentiation media, measured at Day 5, cells display the highest glycolytic capacity at 22% above basal rate. This capacity is significant compared to the glycolytic capacity of cells harvested at Day 2 and Day 10 (*P* < 0.05, *n* = 8) suggesting that oxidative phosphorylation also is important at Day 5 when cells have been induced to differentiate to definitive endoderm in the endoderm differentiation media.

### 3.4. Oxidative Phosphorylation during Differentiation along the Hepatic Lineage

**Figure 4:**
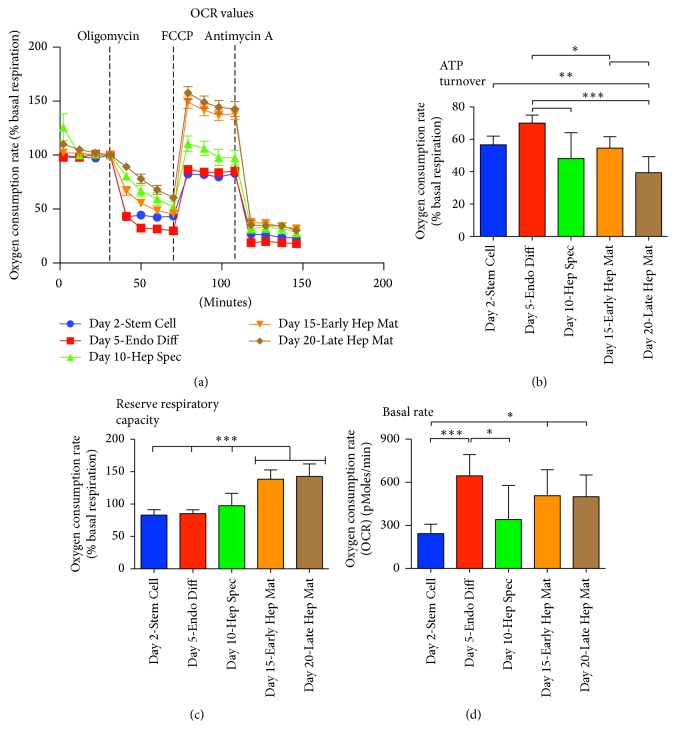
Measurement of oxygen consumption rate at five successive time points in the course of BG01V/hOG (hESC) differentiation along the hepatic lineage. (a) Parameters of oxidative phosphorylation were determined according to rate of oxygen consumption after addition of oligomycin, FCCP, and antimycin A. (b) ATP turnover was measured as percentagewise decrease of oxygen consumption after addition of oligomycin. (c) Reserve respiratory capacity was measured as percentage of basal respiration, after addition of FCCP. (d) Basal respiration measured in pMoles oxygen consumption per minute (^*∗*^*P* < 0.05, ^*∗∗*^*P* < 0.01, and ^*∗∗∗*^*P* < 0.001; *n* = 8; error bars indicate SD).

Throughout the differentiation regimen, cellular ATP turnover and reserve respiratory capacity were determined as attributes of oxidative phosphorylation. Measurements of ATP turnover rates revealed that, at all time points investigated in the course of hepatic differentiation, 40–70% of oxygen was utilized by oxidative phosphorylation. The maximal oxygen consumption rate was present at Day 5 upon differentiation in definitive endoderm induction media and was significantly higher than the following time points (*P* < 0.001–0.05. *n* = 8, Figures 4(a) and 4(b)). Lowest consumption was measured at Day 20 when cells had been cultured in hepatocyte maturation media for 10 days (Figures 4(a) and 4(b)). This demonstrates activity of the oxidative phosphorylation throughout all stages of differentiation. When looking at basal rate measured during the course of hepatic lineage differentiation, lowest levels measured were at Day 2 (Stem Cell) and highest ones were at Day 5 (Endo Diff), Day 15 (Early Hep Mat), and Day 20 (Late Hep Mat) (Figure 4(d)). The basal rates are presented as nonnormalized levels; however the cell count of each processed stage was 115.000 ± 10.000 cells per well (Figure 2(c)).

Reserve respiratory capacity is a measure for the difference between ATP produced by oxidative phosphorylation at basal and that at maximal activity. Through the differentiation regimen, no reserve respiratory capacity was detectable in cells until Day 15 when they had been incubated in hepatocyte maturation media for 5 days. At this time point the reserve capacity was at 138% of basal respiration and significantly higher than the previous three time points investigated (*P* < 0.001, *n* = 8) (Figures 4(a) and 4(c)). Prior to Day 15, the reserve respiratory capacity was lower than the basal respiration. This indicates the presence of very sensitive mitochondria, and we argue that the drop in respiratory capacity is caused by mitochondrial stress induced by treatment with oligomycin and the discrete mechanical force exerted by the XF24 Extracellular Flux Analyzer during measurements of bioenergetics. However, our data suggest that, in cells harvested at Day 2, Day 5, and Day 10, reserve respiratory capacity is completely absent. A significant increase of reserve respiratory capacity is seen upon incubation in hepatocyte maturation media at Day 15 and Day 20.

## 4. Discussion

Mitochondrial dysfunction affecting stem cells has, at least in mice, been related to premature aging [1, 3]. This underlines the importance of a detailed understanding of the role played by mitochondria in stem cell differentiation. In this study, the bioenergetics and mitochondrial network were evaluated at specific time points in the course of stem cell differentiation along the hepatic cell lineage using the human embryonic stem cell line BG01V/hOG.

The adult mature hepatocyte is rich in mitochondria and shows an extraordinary capacity to participate in liver regeneration. The physiologic phenotype of an aging liver is associated with a decreased ability of liver regeneration [19] and a decline of both mitochondrial DNA copy-number and level of oxidative phosphorylation [20]. A decrease of these two mitochondrial properties has been shown to markedly reduce the regenerative abilities of the liver, suggesting a direct link between mitochondrial fitness of hepatocytes and regenerative abilities of the liver [21].

From the rate of glycolysis determined in this study, it is evident that glycolysis is predominant through the entire course of hepatic lineage differentiation, reaching peak level upon incubation in hepatic specification media in what can be argued to be hepatic progenitor cells and lowest level upon incubation in hepatocyte maturation media in what can be reasoned to be hepatocyte-like cells. This is in accordance with the literature stating that glycolysis is the sole provider of ATP in stem cells [6, 22]. Nevertheless, throughout all investigated time points in the course of differentiation, we can demonstrate a presence of oxidative phosphorylation, where an ATP turnover is evident. However, a reserve respiratory capacity only becomes significant at Day 15 and Day 20 upon incubation in hepatocyte maturation media.

When oxidative phosphorylation is inhibited by the ATP synthase inhibitor oligomycin, a compensatory increase of glycolysis may ensue. This increase is defined as the glycolytic reserve capacity. Cells at Day 2 and Day 10 do not display any glycolytic reserve capacity indicating that the oxidative phosphorylation measured at these two time points is not needed for ATP synthesis. Oxidative phosphorylation yields other substrates than ATP. The flavoenzyme dihydroorotate dehydrogenase is a rate-limiting enzyme involved in the de novo synthesis of pyrimidines (reviewed in [23]). The function of the enzyme is linked to the electron transport chain, and the de novo synthesis of pyrimidines is therefore reliant on an active oxidative phosphorylation. It is therefore possible that one role of oxidative phosphorylation demonstrated in stem cells (Day 2) and cells specified to hepatic progenitors (Day 10) is to produce pyrimidines and derived substrates rather than supplying ATP to the cell.

In this study the mitochondrial network was demonstrated to expand once cells had been specified to hepatic progenitors (Day 10) displaying maximum volume upon maturation to hepatocyte-like cells (Day 15 and Day 20). This expansion was accompanied with an increase of reserve respiratory capacity upon incubation in hepatocyte maturation media at Day 15 and Day 20. These last two time points in the differentiation regimen were also characterized with markedly decreased levels of glycolysis and increased levels of glycolytic reserve capacity. Together this highlights the importance of oxidative phosphorylation at the period of early hepatic maturation and emphasizes the transition from hepatic specification to hepatocyte maturation as a period where differentiating cells are potentially more prone to mitochondrial dysfunction than at other stages of differentiation.

Interestingly, we show that cells upon induction in endoderm differentiation media and harvested at Day 5 have an intermittent increase of glycolytic reserve capacity. This is coupled with a basal rate comparable to that of cells incubated in hepatocyte maturation media and harvested at Day 15 and Day 20 and high levels of ATP turnover. As the mitochondrial network at Day 5 still shows tight perinuclear localization and as cells harvested at time points before (Day 2) and after (Day 10) do not display any glycolytic reserve capacity it is tempting to speculate that such an intermittent dependency on oxidative phosphorylation could be a safeguard to allow only differentiating stem cells with functional mitochondria to progress along the course of differentiation. A similar safeguard is hypothesized in fertilized oocytes [24].

Mitochondrial dysfunction of stem cells severely impacts self-renewal capacity and in turn has the potential to result in a progeroid phenotype [3]. Inadequate oxidative phosphorylation has the potential to affect cells through decreased production of ATP, by altering levels of mitochondrial produced ROS functioning as second-messenger molecules and by depleting levels of NAD+ (reviewed in [23]). In turn this has been hypothesized to result in stem cells showing increased sensitivity to stress factors or stem cells that are detained at a specific differentiation stage [22, 25]. Despite all of the work conducted, the exact mechanism linking mitochondrial dysfunction to progeroid stem cells has not yet been described, which is most likely due to the very dynamic nature of differentiating stem cells. In this study, we have described the bioenergetics of a hESC line differentiating along the hepatic lineage and we identified the transition between hepatic specification and hepatocyte maturation as a period, where mitochondrial function is especially important. Our results will contribute to elucidating the relationship between mitochondrial fitness, stem cell health, and aging.

## Figures and Tables

**Table 1:** Antibodies and their origin and dilutions used in the indirect immunofluorescence analyses.

Antibody	Dilution	Company	Catalog number	Secondary antibody (1 : 400)
SOX17	10 *µ*g/ml	R&D Systems	AF1924	Alexa Fluor® 594 donkey anti-goat, A11058
HNF-4*α*	1 : 50	Santa Cruz Biotechnology	SC-6556	Alexa Fluor 594 donkey anti-goat, A11058
AFP	1 : 7000	Dako	A0008	Alexa Fluor 594 donkey anti-rabbit, A21207
CK19	1 : 50	Dako	M0888	Alexa Fluor 594 goat anti-mouse, A21125
EpCAM	1 : 200	Dako	M0804	Alexa Fluor 594 goat anti-mouse, A21125
ALB	1 : 400	Dako	A0001	Alexa Fluor 594 donkey anti-rabbit, A21207
COX IV	1 : 100	Abcam	Ab97770	Alexa Fluor 594 donkey anti-rabbit, A21207
